# Surgery

**Published:** 1912-09

**Authors:** T. Carwardine


REFERENCES.

### SURGERY.

**The clinical aspects and treatment (early and late) of acute perforating ulcers of the stomach and duodenum.**—It may be profitable to the readers of the *Journal* to present a summary of a very exhaustive statistical paper by Ellsworth Eliot on this subject which has appeared recently.[1] The clinical features are important to the practitioner, and the best procedures equally so to the surgeon. The strides have been

[1] *Ann. Surg.*, 1912, lv. 546, 689.

enormous, for some twenty years ago, when the British Medical Association met in Bristol, only half a dozen recoveries had been reported, and it is scarcely thirty years since Mikulicz made the first surgical attempt to save such a case.

Mistakes still occur in **diagnosis,** for it is not always easy; therefore the more prominent symptoms may be considered.

The *history* is that of chronic dyspepsia, but occasionally perforation occurs without any previous warning whatever.

*Pain* is the most constant symptom: without warning, it is sudden and severe in the epigastrium; and at first, if not later, it is accompanied by the most constant and valuable sign of *muscular rigidity* and *tenderness,* the upper segments of the recti muscles being prominent in a retracted, hard epigastrium, with *thoracic respiration,* one or other costal arch being more resistant according to the gastric or duodenal origin. Disappearance of *liver dulness* and *dulness in the flanks* are variable and uncertain signs.

In the **differential diagnosis** the most common confusion is with *appendicitis,* in which the temperature is commonly raised, and in which the signs point rather to the lower half of the abdomen. On operation the distinction is easy by noting the character of the fluid and the condition of the appendix. *Acute cholecystitis,* especially with perforation of the gall-bladder, may give rise to greater difficulty, but operation will decide. *Acute pancreatitis* may simulate either gastric or duodenal perforation, but the fluid is dark or blood-stained, and there may be fat necrosis. Rarer difficulties are with *intestinal obstruction, ectopic gestation,* and *renal calculus.*

It should not be forgotten that ulcers and even perforation may be multiple, and therefore overlooked in part. Statistics point to the desirability of closing the perforation in all cases, and not employing any form of tampon; also to the fact that simple suture of a perforation is quite as safe as excision of the ulcer, and that it is followed by healing of the ulcer. In the early cases peritoneal drainage may not be necessary, but in the later cases it is essential, especially in the subphrenic spaces, for subphrenic abscess is the most frequent and fatal post-operative complication. It is interesting to find in America preference for drainage by soft rubber tubes, as is the practice in this country.

The question of gastro-enterostomy at the time of operation is important. It can generally be omitted with advantage unless the indications for its performance are really strong. The giving way of sutures is not common, and forebodings of subsequent stenosis are not generally realised. Gastro-enterostomy probably diminishes the danger of perforation of co-existing ulcers, but does not entirely prevent it; and it cannot be relied upon to prevent subsequent hemorrhage, for several fatalities have occurred from this cause even after an added gastro-enterostomy. On the whole, a simultaneous gastro-enterostomy is best omitted, although there are different views on this subject, and corresponding varieties of practice amongst surgeons. The best argument for simultaneous gastro-intestinal anastomosis is that in some thirty-eight published cases a subsequent gastro-enterostomy has been necessary for the relief of symptoms, and these happen to include two of my own published cases.[2]

In the original paper the following tables of published cases are given: Suture, followed by secondary gastro-enterostomy, with relief (38). Ditto, without relief (2). Suture followed by perforation of co-existing ulcers within a month (14). Suture with gastro-enterostomy followed by ulcers within a month (3). Suture followed by remote perforation (8). Suture with gastro-enterostomy followed by remote perforation (3). Suture followed by hemorrhage (10). Suture with gastro-enterostomy followed by hemorrhage (3). Gastro-enterostomy for ulcer followed by perforation within one month after operation (13). Gastro-enterostomy for ulcer followed by hemorrhage (29). Gastro-enterostomy for ulcer followed by cancer (3). Gastro-enterostomy at time of closure of perforation (89 with recovery, 30 with death). In all there were 75 cases in which gasto-enterostomy plus the closure of a perforation would have been of advantage, in the hands of a rapid and experienced operator.

It may be noted that the writer has tabulated the results of gastro-enterostomy for duodenal and gastric ulceration, apart from perforation and comprising one thousand cases from various sources, and the percentage of good results in each class varies from 70 to 80 per cent. In the majority of cases in which simple suture has been successfully performed for perforation the patients have been permanently improved or cured of the gastric symptoms quite apart from gastro-enterostomy.

To the valuable paper of which the above is a summary the writer has added an extensive bibliography, comprising over a hundred references.

T. CARWARDINE.

### OPHTHALMOLOGY.

The subject of **miner's nystagmus** has been very much to the fore of late. Now that it has been made one of the scheduled industrial diseases, the colliery proprietors as well as the Government, have been making special inquiries and investigations into its cause and cure, if there is such a thing as a cure. Here in Bristol one does not see quite so many cases as formerly, although cases are not infrequent. A very interesting

[2] *Lancet*, 1910, i. 239.

